# Management of pandemic or large-scale emergencies in Germany with a focus on the current and potential role of university schools of dentistry: Can it help in COVID-19 time?

**DOI:** 10.1186/s42269-020-00427-4

**Published:** 2020-10-02

**Authors:** Marcus Schiller, Marijatta Pilette, Björn Rahlf, Constantin von See, N.-C. Gellrich

**Affiliations:** 1 Department of Craniomaxillofacial Surgery, Hannover Medical School, Carl-Neuberg-Straße 1, 30625 Hannover, Germany; 2 Department of Dentistry, Danube Privat University, Steiner Landstrasse 124, 3500 Krems-Stein, Austria

**Keywords:** COVID-19, Pandemic, Disasters, Emergency medicine, Mascal

## Abstract

**Background:**

The study presented here systematically examines the potential involvement of dental, oral and maxillofacial centres (ZMK) in the management of pandemia or in large-scale emergencies. It looks at available material and infrastructural resources and how they can be brought to bear in such incidents or situations. The aim was to gain an initial scientific overview of how ZMK can potentially contribute to the handling of a pandemia or mass casualty (MASCAL) situation in terms of available resources as well as their location within the hospital as a whole and their integration into the existing infrastructure. The study was conducted on the basis of a questionnaire consisting of 70 individual questions, which was sent to all universities in Germany that offer a course of study in dental medicine. The responses were then statistically evaluated.

**Results:**

The study outlines the current status of ZMK and discusses what could be an important component of emergency medical care in the overall hospital context.

**Conclusion:**

The involvement of ZMK—with their own resources and existing infrastructural links to the hospital as a whole—could lead to faster and more effective patient treatment in the event of a pandemic or MASCAL situation.

## Background

The increasing threat of international terrorism has shifted public focus in Germany onto the question of how large-scale emergencies can be managed.

After the 9/11 terrorist attacks in New York, the Standing Conference of Federal and State Ministers and Senators of the Interior decided to critically review civil protection and disaster control policies (Simon and Tepermann 2001; Cook 2001).^1^ As a result, hospitals were also forced to take a closer look at this issue and to re-evaluate existing emergency response plans, especially after the recent terrorist attack on a Christmas market in Berlin. One question that needs to be answered is how to sensibly involve dental medical centres in existing emergency concepts.

So far, there have been widely differing views on this subject at the various hospitals, although there already are several German and international studies available on the potential integration of such centres (Pahor 1992; Sakr 2000; Mitchell 2008). That is because in general, each dental treatment unit (i.e. dentist’s chair) could be considered a small operating table in its own right. After all, it provides an opportunity for surgical hand disinfection, a small surgical light, a suction device, monopolar electrocoagulation and, above all, various ways to position the patient, including the shock position. Examinations and surgical treatments such as wound care, splinting or other emergency treatments are possible.

Taking into account previous studies, the aim of this study was thus to systematically investigate the current rate and potential increase in integration of dental medical centres at university hospitals in the emergency response plan of the hospital as a whole.

For this purpose, a specifically developed questionnaire was used to survey the structures and resources of the dental medical centres at German university hospitals. Their potential involvement in providing emergency medical treatment in case of large-scale emergencies is illustrated using Hannover Medical School (MHH) as an example. Previous contingency plans for a mass casualty scenario had foreseen the fire service, Germany’s Federal Disaster Relief Agency (THW) and the Red Cross setting up and operating treatment stations at the outpatient clinic of the MHH to support the hospital. The option of falling back on the dental treatment stations of the dental medical centre is currently not included.

### Previous findings

Emergency medicine as we know it today is a relatively new medical discipline. Its history can be traced back to lessons learned in military campaigns that were translated into principles for the rescue and evacuation of casualties. It was not until after World War II that civilian emergency medicine became truly established (ambulances, triage, etc.).^2^ Härtel stated in 1920 that physicians who worked in other specialties during times of peace had to get used to thinking and acting as surgeons in times of war (Robertis et al. (2017)).

### The path towards civilian emergency medicine

The way ambulance services in Germany are organised today is a result of the adaptation of military principles, the further development of medical knowledge and increasing regulation (Skandalakis et al. 2006).

During World War I, emergency rescue was the responsibility of the Red Cross and the fire services. After World War II, the emergency rescue services were shaped by the occupying powers and the different occupation zones. Rutherford suggested that the order of evacuation should depend on the pattern of injury in the different triage categories.^3^

## Description of the German disaster control organisation

### Legal situation in the Federal Republic of Germany

On the federal level, civil disaster control tasks fall into the remit of the Federal Ministry of the Interior and are allocated to the Federal Office of Civil Protection and Disaster Assistance. This office is a higher federal authority and supports the supreme federal authorities in uniform civil defence planning. In case of a hazard or emergency situation, crisis staffs at the Federal Ministry of the Interior assume coordination tasks (Niska and Shimizu 2011). In Germany, 95% of medical assistance delivered in an emergency situation is provided by non-governmental organisations.^4^^,^^5^ A system of fast response units has been established, which are mainly employed for preclinical tasks (Sakr 2000).^6^^,^^7^

### The Länder (German federal states)

Disaster control in Germany reflects the German federal system as federal law assigns certain tasks to the Länder [Article 73 (1) of the Basic Law]. However, the Federation also makes recommendations and cooperates with the Länder [Article18 (1) of the Civil Protection and Disaster Management Law]. This is especially the case in large-scale emergency situations or emergency situations of national significance (Niska and Shimizu 2011). In order to improve joint coordination and practice, the Federal Minister of the Interior and the ministers of the interior of the Länder decided in 2002 to conduct a national crisis management exercise.

#### Lower saxony

Over 180 hospitals in Lower Saxony provide medical care for the state’s population. In the event of an emergency, the number of patients they will treat will exceed normal capacity. In this context, a contingency plan may be important to facilitate an appropriate response. According to Sefrin et al., a working emergency response plan is a prerequisite for extending the treatment capacities of every hospital in an emergency (Schenk 2008).

Allocating patients in successive waves may counter clinical overload (Adams and Tecklenburg 2007). This plan of admitting patients in “waves” makes it possible to maintain the hospitals’ ability to act, even though requests for treatment capacity can no longer be accommodated.

The tool used by the Länder for adapting to actual needs is the so-called hospital plan. This plan provides the basis for ensuring requirement-oriented support of the population with respect to the hospitals needed according to their location, specialties, number of beds and functional units (Niska and Shimizu 2011; Mistovich et al. 2013). The state of Lower Saxony has such an emergency response plan in place.

### Hannover medical school

The Hannover Medical School (MHH) is a well-established university hospital and a supramaximal care hospital. With a capacity of about 1500 beds (as of 2013), the MHH is one of the largest hospitals in Lower Saxony. Together with the University Medical Centre Göttingen, the MHH is one of two hospitals in Lower Saxony to feature a university dental medical centre and offer a course of study in dental medicine. The 2015 hospital plan of Lower Saxony states that the number of beds assigned to oral and maxillofacial surgery is equivalent to 0.3 inpatient beds per 10,000 inhabitants (Adams and Tecklenburg 2007).

### Background knowledge

A credo of emergency medicine is that each patient should be provided with individual care as quickly as possible, but not past the point where, in the case of a large number of casualties, the treatment of that individual patient would have a disproportionate negative effect on the prognosis of others.

Forecasts about the type of patients admitted to hospital as well as their patterns of injury and time of arrival are mostly based on the nominal analysis of patient numbers.

## Methods

The population of the study consisted of 28 hospitals.

**Fig. 1:**
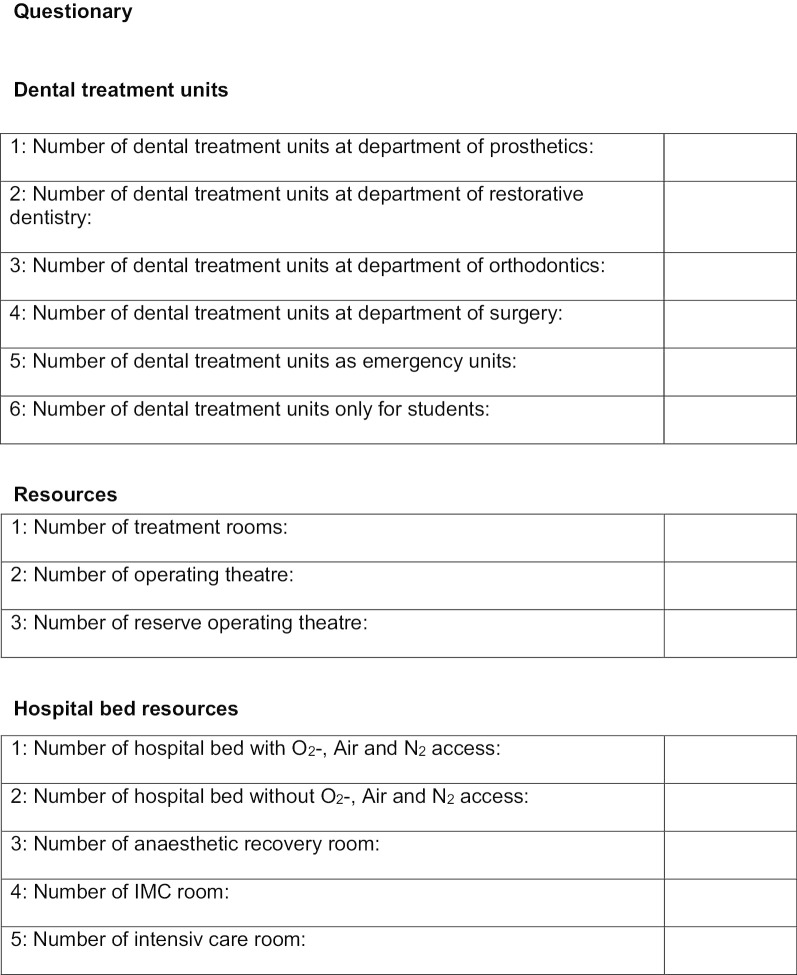
Questionary

Questionnaires were sent out to the following university hospitals with dental medical centres and/or dental student training (Fig. 1):

**Table:** 

Aachen	Berlin	Bonn	Düsseldorf	Dresden
Erlangen	Frankfurt	Freiburg im Breisgau	Giessen	Göttingen
Greifswald	Halle (Saale)	Hamburg	Hannover	Heidelberg
Jena	Kiel	Cologne	Leipzig	Mainz
Munich	Münster	Regensburg	Rostock	Saarbrücken
Tübingen	Ulm	Würzburg		

### Data processing and statistical method

#### Digital data acquisition

For digital data acquisition, Microsoft Excel for Mac 2011 (14.2.1 update) was used. Of each hospital surveyed, 70 individual data items were collected in the Excel file. For statistical processing of the data, IBM SPSS Statistics 19 (IBM Deutschland GmbH) was used.

### Ethics and consent to participants

In the run-up to the study, ethics commission at the Hanover Medical School was asked. This study does not require an ethics vote, as the study is purely anonymous.

## Results

Of the 28 university hospitals-based dental medical centres that were invited to participate in the study, 71.4% returned the questionnaire.

### Instruction rooms

Most dental medical centres feature instruction rooms (88.0%). In 14 (60.9%) of these centres, such rooms have separate entrances with direct access to the outside. Only 30.4% of them lack access to the outside area.

### Sterile material

Sterile processing is carried out in three different places: at the central sterile processing department of the entire hospital, at the central sterile processing unit of the dental medical centre or locally, i.e. at the individual departments of the dental medical centre.

If only the central sterile processing department (*N* = 4) or a combination of that department and the central sterile processing unit at the dental medical centre (*N* = 4) is used, transportation/supply of sterile items is possible 24 h a day.

If the hospital provides transportation services for patients on a 24-h basis all year round, sterile material will also always be transported/supplied 24 h a day (*N* = 18). Of the hospitals that do not provide 24-h transportation/supply of sterile items, 50% did not provide a patient transportation service either.

### Treatment options

On average, the dental medical centres feature a total of 82.5 dental treatment units and 4.9 surgical rooms, which fall into the categories of minor surgery rooms, emergency operating theatres and operating theatres.

**Fig. 2:**
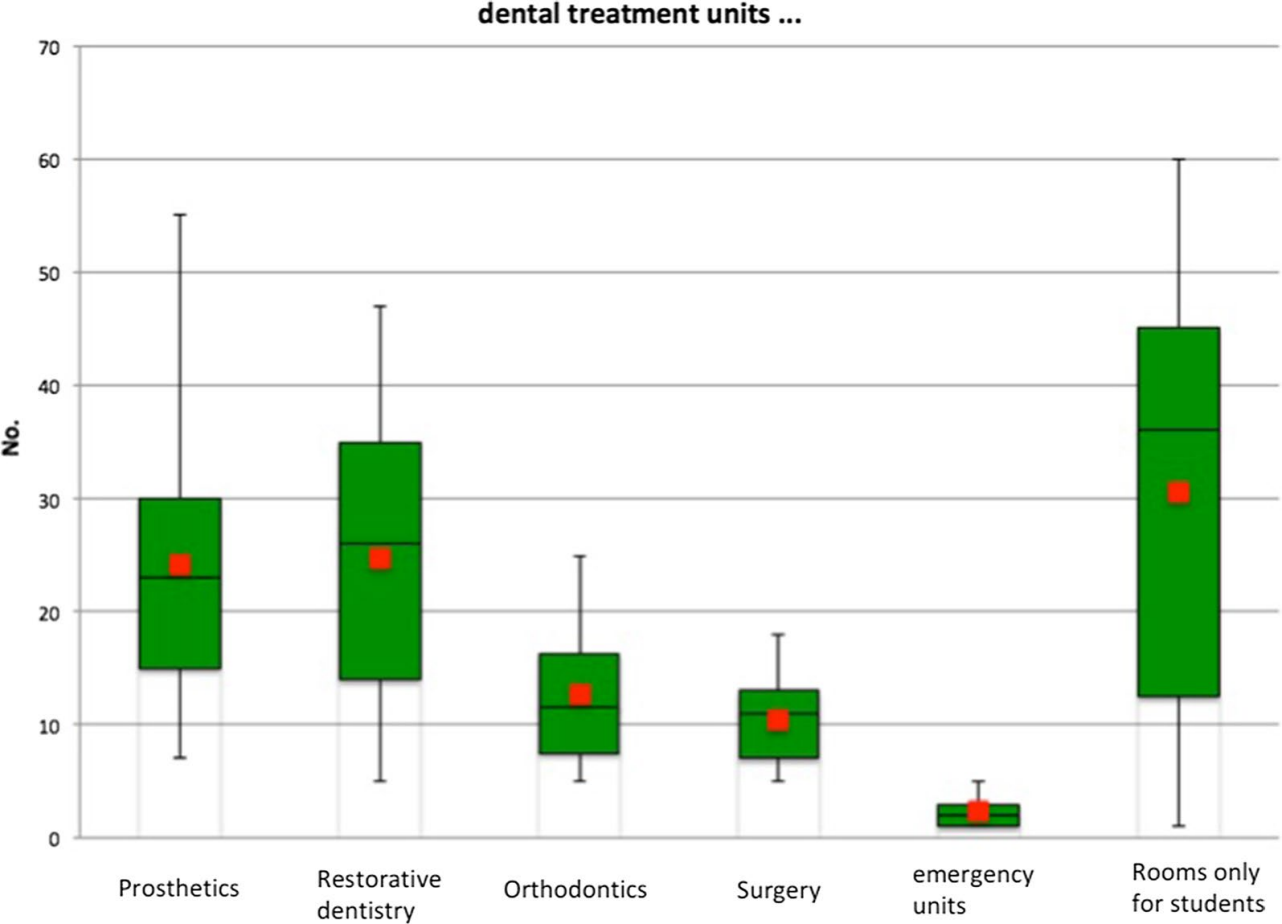
Numbers of available minor surgery rooms, operating theatres, emergency operating theatres

In dental treatment centres equipped with dental treatment units, the numbers are: (Fig. 2).

**Fig. 3:**
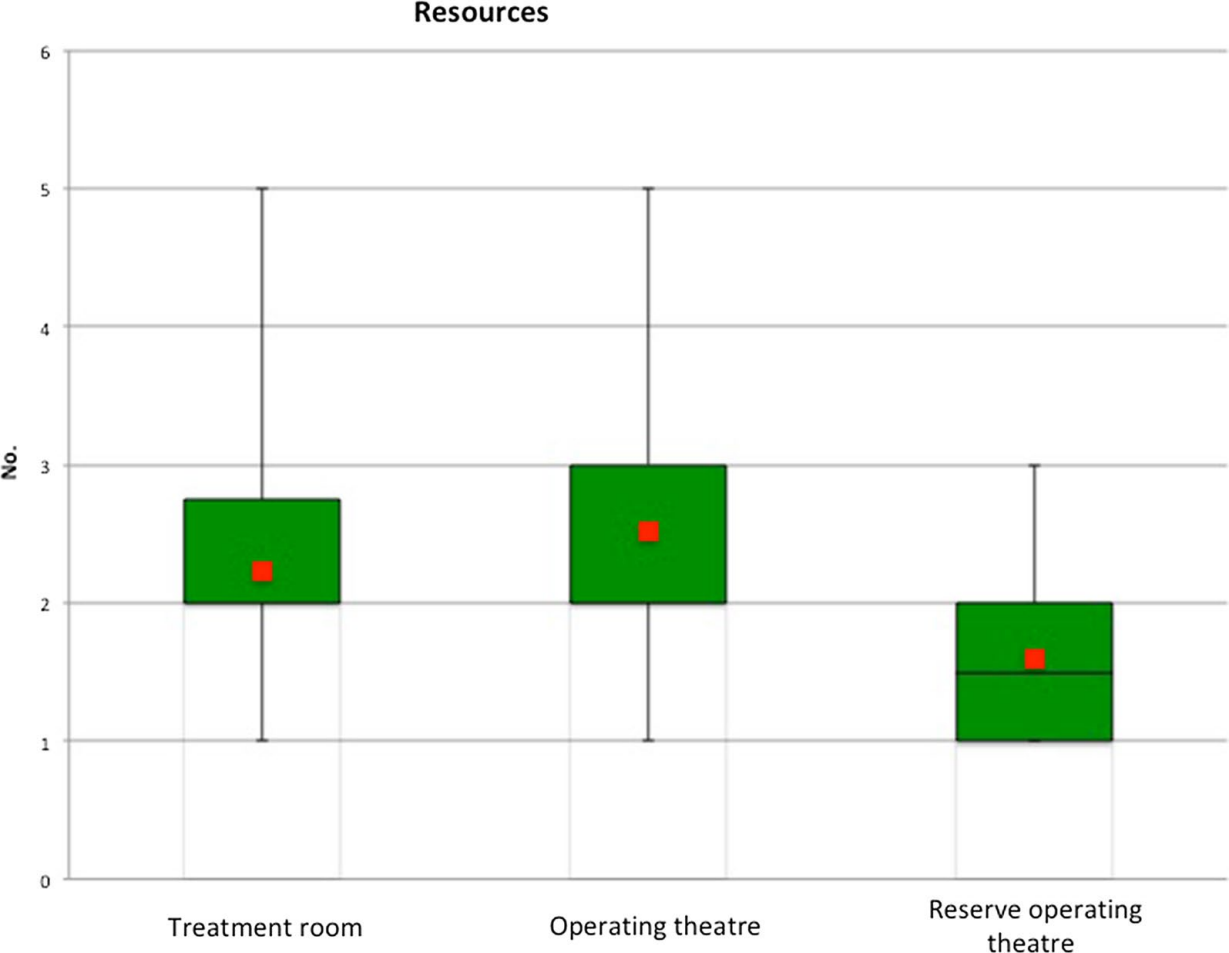
Numbers of ward beds, recovery room places, IMC and intensive care beds

The number of minor surgery rooms, operating theatres and emergency operating theatres was also determined. The graph illustrates the results (Fig. 3).

### Bed capacities

An average of 25.1 ward beds are available. The questionnaire differentiated between ward beds with and without gas supply. The parameters are listed in Table 1.

**Table 1:** Hospital bed resources

	*n*	Average	Minimum	Maximum	Standard deviation
Hospital bed with O2-, N2- and air connection	23	20.96	0	42	14.2
Hospital bed without O2-, N2-, compressed-air connection	23	9.5	0	38	9.5
Anaesthetic recovery room	23	2.3	0	8	2.3
IMC	23	1.7	0	4	1.7
Intensive care	23	0.4	0	3	0.9

On average, dental medical centres have 2.4 recovery room places and 1.0 intermediate or intensive care places (IMC places).

Four dental medical centres are equipped with intensive care capacities. A maximum of three intensive care beds are available in 4.5% of all dental medical centres. Two such beds are available in 13.0% of the centres. This results in an average capacity of 0.4 intensive care beds at dental medical centres.

The ratio of surgical room capacities to bed capacities is 1:7 (one operating theatre per seven ward beds). The ratio between operating theatre capacity (4.9) and recovery room places (2.35) is approximately 1:2.

### Digital networking

Upon investigating the dependency on information technology (IT), we found that a digital record exists in 36.0% of all hospitals, including dental medical centres.

## Discussion

The number of patients to be treated in a mass casualty event is always difficult to estimate (Mistovich et al. 2013; Rutherford 1989). It depends not only on the total number of people affected by the event but particularly on the number of people with injuries that see them classified as triage categories I–III. A comparison of the German Eschede train disaster with the terrorist attacks in Madrid in terms of victims shows that the number of injured who reach a hospital does not have a linear correlation with the overall number of people affected. In Eschede, only 50% of casualties were alive upon arriving at a hospital for treatment, compared with 90% of victims in Madrid (Turégano-Fuentes et al. 2008). Insufflation anaesthesia is required for almost all patients of triage categories I and II. Of the total number of patients to be expected, 60% will fall into these two categories. At least 20% of trauma patients classified as “category red” are in need of life-saving emergency surgery. For critically injured patients, surgical capacity is one of the decisive bottlenecks. Analyses of the surgeries performed after the Madrid bombings have shown that out of the 124 operations carried out within the first 24 h, 17 were maxillofacial surgeries. About 10% of these injuries were pure fractures of the jaw and facial bones, while around 66% affected the face, head and neck area. Of all seriously injured category I patients, approximately 26% require surgical interventions in the region of the head and neck.

Patients with minor injuries can be expected to make up a proportion of 40%. Adams has in different publications already suggested having these patients treated at a dental, oral and maxillofacial clinic by staff of the dental medical centre. Triage category III patients can usually be treated under local anaesthetic. Treatment under local anaesthetic is part of the standard treatments routinely performed by dentists (Pahor 1992; Daubländer 2012). They have the required facilities and equipment at their disposal, and treating their patients in a dentist’s chair is common practice for them. This resource, which is available in relatively large numbers, should thus be considered for use in major emergency situations. In that case, such facilities and the dental treatment units would mainly be used for the treatment of triage category III patients. This would involve the organisational integration of the staff and in some cases the students at dental medical centres, who would cooperate with doctors and assistant personnel in a multidisciplinary approach. A dentist’s chair can generally also be used for the treatment of regions other than the head and neck area. An average of 86 dental treatment units are available, which means that 86 treatment stations are available for triage category III patients. These units are spread all over Germany according to the distribution of universities for dental, oral and maxillofacial medicine. In addition, dental medical centres lend themselves to providing rooms for crisis staffs or families as well as for pastoral care, etc. This workload sharing within hospitals equipped with a dental medical centre would make it possible to more efficiently use the resources for triage categories I and II at the main hospital. If the condition of a triage category III patient deteriorates during treatment and a more severe triage category needs to be assigned, the traditional surgical and monitoring facilities within the dental medical centres can be incorporated across the specialties.

## Conclusion

The objective of this study was to investigate the existing and future potential integration of dental, oral and maxillofacial clinics into the emergency concepts of hospitals as a whole. The data obtained were statistically evaluated and analysed. The majority of dental medical centres in the area surveyed were found to not be included in the emergency concepts of university hospitals.

Of the 18 dental medical centres that are in fact included in emergency concepts, only two-thirds were able to provide details on the exact nature and extent of this involvement. We thus conclude that resources for patient examination, treatment and admission are generally available but not used to their full extent. In terms of these resources, we were able to establish the numbers of dental treatment units (average 82.5), minor surgery rooms (average 2.8), operating theatres (average 3.0) and ward beds (average 25.1).

We further conclude that dental medical capabilities in Germany are insufficiently used as a potential resource in case of an emergency. There are doubts about the integration of dental (assistant) personnel. However, the geographical distribution of existing dental medical centres is an additional positive aspect in terms of their potential involvement in case of large-scale emergencies.

In the current situation, hospitals are flooded with a large number of patients. The focus of the hospitals is on the treatment of emergencies and especially on the treatment of COVID-19 patients.

Additional places are being created for triage, either by setting up additional treatment places or by putting up tents. The use of the resources of dental medical centres to relieve the main clinics should be included in the considerations. Dental medical centres offer a large number of possibilities for the initial treatment and/or treatment of patients with mild to severe diseases.

The integration of dental medical centres, with their own resources and existing infrastructure connections to the hospital compound, could facilitate quicker and more efficient treatment in a mass casualty event. In such emergency situations, physicians and other non-dental medical personnel could take up work at the dental medical centres, and their dentist colleagues who work in hospitals and in outpatients setting could also be involved. The necessary statutory provisions would first have to be established, however.

The USA sets a positive example in this regard. Constant further development of the task spectrum of dentists as part of mass casualty planning and the creation of a clear statutory framework ensure that all capabilities are exploited to their full potential. Through extended training in the field of emergency medicine as students, dentists are thoroughly prepared for their future tasks and thus able to provide a real contribution to casualty care in large-scale emergencies.

## Data Availability

The datasets used and/or analysed during the current study are available from the corresponding author on reasonable request.

## Abbreviations

MHH: Hannover Medical School
THW: Germany’s Federal Disaster Relief Agency
GmbH: Limited liability company
IMC: Intermediate care

## Footnotes

1 Cabinet bill by the des Ministry of the Interior of Lower Saxony, *Eckpunkte für die Errichtung eines Kompetenzzentrums für Großschadenslagen im Niedersächsischen Innenministerium.* 2003.

2 "What is Emergency Medicine?". Yale School of Medicine. Archived from the original on 19 November 2010. Retrieved 18 March 2011.

3 Parliament. Human casualties who sustain injuries leading to death less than 30 days after the accident. (This is the usual international definition, adopted by the Vienna Convention in 1968.)

4 NAEMT, *Präklinisches Traumamanagement*. 2009.

5 https://www.cilip.de/terror/imk-11-2011.pdf.

6 Anlage 2 zu Beschluss Nr. 26. der 174. Sitzung der Ständigen Konferenz der Innenminister und -senatoren der Länder vom 8. Juli 2004 in Kiel.

7 Niedersächsischer Landtag, *Drucksache 15/974*. 2004.
